# Competition Stress Leads to a Blunting of the Cortisol Awakening Response in Elite Rowers

**DOI:** 10.3389/fpsyg.2019.01684

**Published:** 2019-07-18

**Authors:** Douglas MacDonald, Mark A. Wetherell

**Affiliations:** ^1^Psychobiology Research Group, Department of Psychology, Northumbria University, Newcastle upon Tyne, United Kingdom; ^2^Scottish Canoe Association, Edinburgh, United Kingdom

**Keywords:** stress, anticipation, competition, rowers, cortisol awakening response

## Abstract

**Background**: Anticipation of forthcoming demands is often met with biological up-regulation, for example, levels of the stress hormone cortisol are typically elevated immediately prior to an anticipated event. Similarly, the cortisol awakening response (CAR), a surge in cortisol in the period following waking, is elevated on days of anticipated demand and this is viewed as an adaptive response in the preparation for challenge. This study assessed the effects of competition as an anticipated challenge in elite rowers.

**Methods**: Elite rowers (*N* = 8) were assessed during two training and two competition weekends. Each assessment involved the measurement of self-reported competitive (cognitive and somatic) anxiety and salivary diurnal cortisol across 2 days representing a preparation day prior to either a training or competition day. Competitive anxiety was measured each morning and saliva samples were provided immediately upon waking and 30 min post waking (CAR) and before bed.

**Results:** Self-reported cognitive and somatic anxiety levels were significantly greater during the competition phase compared with training. Additionally, levels of cognitive anxiety were greater on the day of competition compared with the preparation day. CAR magnitude was significantly reduced during the competition phase compared with training; however, there were no differences between preparation and event days.

**Conclusions**: Reduced or blunted CARs are typically observed in chronically stressed populations and are characteristic of burnout and fatigue. While an increased CAR during competition may represent an adaptive response to challenge, blunted CARs and the concomitant increases in competitive anxiety observed here indicate maladaptive responding during a period where maximized functioning is critical.

## Introduction

The anticipation of forthcoming demand is ubiquitous in competitive sport and the period prior to competition is typically accompanied by changes in mood, in particular, increases in competition anxiety. This manifests as cognitive anxiety, characterized by a lack of concentration, disrupted attention, and ruminations about performance, and as somatic anxiety, characterized by self-perceptions of how arousal has affected the body (Martens et al., 1990a). Situations in which an individual anticipates a requirement to react or respond are typically met with increased biological activity, in particular, increases in neuroendocrine and cardiovascular activity, and this biological up-regulation serves to prepare the individual for the forthcoming perceived demand.

For forthcoming events where demand is likely, it is therefore adaptive for resources to be mobilized before the event is encountered in order that the appropriate response can be initiated immediately. This is especially the case where there is a need for rapid responses, and activation of two key physiological systems, the sympathetic-adrenal-medullary (SAM) and hypothalamic-pituitary-adrenal (HPA) axis provide resource to deal with the presenting situation (Hare et al., 2013). For example, activation of the SAM axis leads sprinters to demonstrate cardiovascular up-regulation before the starter’s gun (McArdle et al., 1967; Inui, 1987) and this facilitates immediate physical exertion at the start of the race. Cortisol, secreted by the HPA axis, is an energizing hormone responsible for mobilizing activities directed toward responding to demand or threat. When faced with a demanding situation, acute up-regulation of cortisol increases the release of glucose and stimulates the sympathetic nervous system providing resource to deal with the threat and maintain homeostasis (Wetherell et al., 2006). Accordingly, higher levels of cortisol have been observed immediately before and during competition compared with practice rounds in golf (McKay et al., 1997), and immediately before a judo competition compared with resting days (Salvador et al., 2003). A recent review concluded that cortisol levels are elevated in athletes prior to competition and that levels are greatest when sampled closer to the start of competition (van Paridon et al., 2017). This may provide evidence of an adaptive role for biological up-regulation to enable resources immediately prior to demand.

The nature of this up-regulation, however, depends upon how the forthcoming event is perceived. The Theory of Challenge and Threat States in Athletes (TCTSA: Jones et al., 2009) demonstrates how this may manifest in the context of sporting competitions. That is, whether forthcoming competition is perceived as a challenge or a threat determines whether athletes respond positively or negatively, respectively. Moreover, this perception can lead to distinct patterns of biological up-regulation. Specifically, perceptions of challenge typically lead to activation of the SAM axis, subsequent increases in cardiac activity and reductions in peripheral vascular resistance, which serve to mobilize resources to facilitate coping. Perceptions of threat, however, are associated with SAM and HPA activation, leading to corticosteroid release without corresponding reductions in vascular resistance. This pattern of responding is not facilitative for coping and has negative consequences in terms of emotions and performance (Jones et al., 2009). The TCTSA therefore suggests that biological up-regulation is determined by the perception of the event, and that this can lead to adaptive or maladaptive psychobiological responding depending on whether the event is perceived as a challenge or a threat.

In addition to changes in response to acute threat, the secretion of cortisol follows a marked diurnal profile characterized by a rapid increase in the 30–45 min immediately following awakening (cortisol awakening response, CAR) and a decline across the day toward lowest levels around midnight (Stalder et al., 2016). Deviations from this typical increase following awakening are characteristic of neuroendocrine dysregulation and associated negative consequences for health and well-being. For example, reduced CARs, typically described as blunted, are associated with fatigue, burnout, and exhaustion (Chida and Steptoe, 2009).

Although in sporting contexts, acute HPA activation is typically associated with perceived threat, there is now increasing evidence that the secretion of cortisol during the CAR period is an adaptive response that plays a role in the preparation for forthcoming demands (Fries et al., 2009; Clow et al., 2010). Increased CARs have been observed during periods of greater uncertainty and workload (Brant et al., 2010); on workdays characterized by greater feelings of stress (Kunz-Ebrecht et al., 2004); and in teachers on the day of an assessed observation (Wolfram et al., 2013). CARs of greater magnitudes have also been observed on the day of a manipulated social and cognitively demanding stressors in ambulatory (Wetherell et al., 2015) and controlled sleep laboratory conditions (Elder et al., 2018). Increased cortisol secretion has also been observed on the days of competitive sports. Elevated levels of cortisol were observed in the morning of dancing (Rohleder et al., 2007), motorcycling, and tennis (Filaire et al., 2007, 2009) competitions, compared with control days, and in the evenings during a 3-day soccer competition compared with sampling prior to the competition (Aizawa et al., 2006). In contrast, no change in cortisol secretion in the hour following awakening was observed 3–7 days prior to and on the day of a martial arts competition (Strahler et al., 2010) suggesting possible adaptation of the HPA axis in the lead up to competition.

Given the potential role of the CAR as a marker of anticipation of forthcoming demand, it may provide a novel insight into the impact of forthcoming competition on neuroendocrine functioning. The current study is therefore the first to assess both the acute and prolonged impact of competition on the CAR. Specifically, the study followed a crew of eight male elite rowers across consecutive weekends of training and competition, assessing competition anxiety and cortisol indices on the day before (preparation) and the day of (participation) a competitive event. It was predicted that levels of competition anxiety will be greater during competition compared with training weekends and on competition days compared with preparation days. Based on studies suggesting that the CAR has an adaptive role in preparing for forthcoming demand, it is predicted that greater CARs will be observed during competition compared with training weekends and on competition days compared with the preparation days.

## Materials and Methods

### Participants

All recruitment and study procedures were granted ethical approval from the Faculty Ethics Committee in line with relevant regulatory bodies. Eight male university rowers (range: 19–23 years; *M*_age_ = 20.62, SD = 1.30) participated in the study. All participants were part of an elite rowing program and were competing at national or international level. Throughout the testing period, the athletes trained under the same program and competed as a team. None of the participants were taking drugs or medication and had no known history of endocrine disorders. Prior to the last weekend of testing, one rower developed an injury which made him unable to compete in the last race and his data from the final weekend are excluded from analyses.

### Measures

#### Salivary Cortisol

The researcher met with all rowers and their coach to detail the requirements of the protocol, engage them with the study goals, and explain saliva collections procedures. Participants collected their own saliva using Salivettes (Sarstedt, Germany). All samples were frozen (−20°C) and assayed using the enzyme-linked immunosorbant assay method (Salimetrics-Europe, UK; intra and inter-assay coefficients <10%). Cortisol values are reported in nmol/L.

#### Competitive Anxiety

The Competitive State Anxiety Inventory (CSAI-2, Martens et al., 1990b) was used to record self-reported levels of cognitive and somatic anxiety. The CSAI-2 comprises 27 items rated on a 4-point Likert scale with higher scores indicating higher levels of each state. It is a well-established tool for the assessment of competition anxiety across a wide variety of sporting contexts and has been previously used, alongside the measurement of cortisol, to differentiate practice and competition in sport (McKay et al., 1997).

### Protocol

Testing took place over 4 consecutive weekends during April and May, incorporating 2 weeks at the end of the training season and the first 2 weeks of the competition season. Each weekend comprised 2 days of testing: during training weekends, these comprised typical training, and during competition weekends, comprised a preparation day (the day before competition), and a participation day (the day of competition). On each testing day, rowers provided three cortisol samples: immediately upon awakening; 30 min following awakening; and before bed. The CSAI-2 was completed each day following the provision of the 30-min sample and prior to training or competition. Training weekends comprised a 5- to 10-km run followed by a 16-km row on day one, and two 10- to 12-km high-intensity rows followed by one 16-km low-intensity session on day two. The first competition weekend was the British Universities’ competition, which comprised eight crews racing in heats, semi-finals and finals (depending on success). The second weekend was the annual inter-varsity race which comprised two crews in a single head-to-head race.

### Treatment of Data

The dependent variables of awakening cortisol, CAR, levels of cortisol prior to bed, cognitive anxiety, and cognitive anxiety were assessed using ANOVAs with repeated measures for each independent variable: Phase (Training versus Competition); Weekend (Practice 1, Practice 2, Competition 1, Competition 2); and Day (Preparation versus Participation). The CAR was calculated by subtracting cortisol levels at awakening from levels at 30 min post waking. All data used in analyses are provided in Data Sheet 1.

## Results

There were no significant differences in levels of cortisol at awakening or prior to bed between phases, weekends, or days of testing (*p* > 0.05). The phase x day interaction demonstrated higher levels of cortisol at awakening on competition days compared with training days; however, this was not significant (*p* = 0.06, *η*^2^ = 0.41).

**Figure 1:**
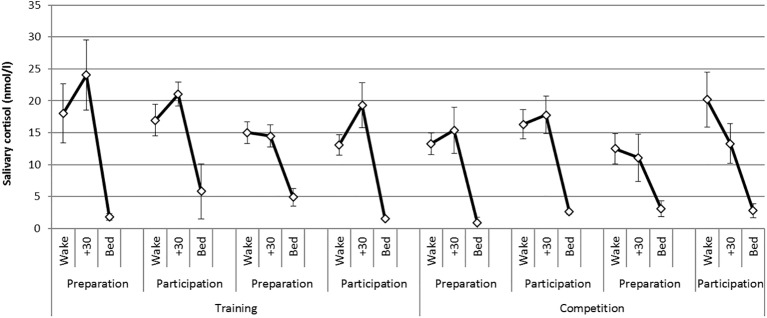
Mean (S.E) diurnal (awakening, +30 min, bed) cortisol profiles on each day of testing across the training and competition weekends.

There was a statistically significant difference in the CAR between phases [*F* (1,6) = 20.1, *p* = 0.004, *η*^2^ = 0.77] where CAR magnitude was greater during the training phase compared with the competition phase. There were no significant differences in the CAR between weekends or days of testing. Figure 1 presents mean diurnal cortisol profiles on each day of testing across the practice and competition weekends. The CAR is represented as the change from levels at awakening to levels at 30 min post awakening.

**Figure 2:**
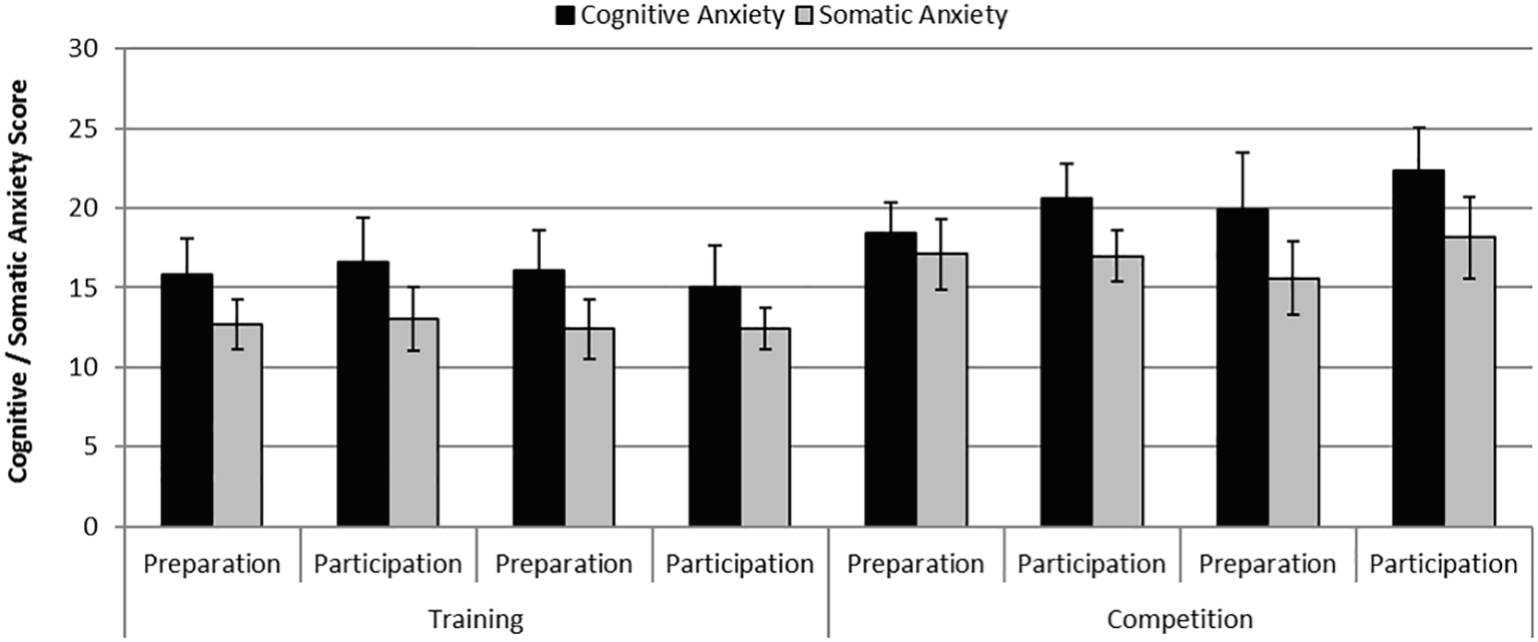
Mean (S.E) self-reported levels of cognitive anxiety and somatic anxiety on each day of testing across the training and competition weekends.

There were statistically significant differences in cognitive anxiety [*F* (1,6) = 8.13, *p* = 0.029, *η*^2^ = 0.58] and somatic anxiety [*F* (1,6) = 7.87, *p* = 0.031, *η*^2^ = 0.57] between phases where anxiety levels were greater during the competition phase compared with the practice phase. Additionally, there was a significant effect of Day on cognitive anxiety [*F* (1,6) = 20.61, *p* = 0.004, *η*^2^ = 0.78] where anxiety levels were greater on the participation day compared with the preparation day. Figure 2 shows mean self-reported levels of cognitive and somatic anxiety on each day of testing across the practice and competition weekends.

## Discussion

In line with predictions, rowers reported significantly greater levels of cognitive and somatic anxiety during competition compared with training, and greater levels of cognitive anxiety on the participation day compared with the preparation day. There were no differences in the CAR between participation and preparation days, but there were significant differences between competition and training. Contrary to prediction, however, greater CARs were not observed during competition, but were significantly blunted during competition compared with training.

Evidence in sporting and non-sporting contexts suggests an adaptive role for increased CARs in preparation for forthcoming demand. In support, increased cortisol has been reported on the mornings of competition compared with control days. In the current study, the magnitude of the CAR, however, was not greater during competition weekends or on the day of competition. This finding is similar to those of Strahler et al. (2010) who observed no increases in the CAR in martial artists in the week-long lead up to a competition, suggesting that elite athletes become accustomed to competitive environments and show neuroendocrine habituation. Reduced psychobiological reactivity to acute stress has also been observed in elite athletes compared with non-trained controls (Rimmele et al., 2007). Adaptation is therefore one explanation for the current findings. That is, elite athletes demonstrate reduced stress responsiveness either as a positive function of repeated exercise or habituation to competition (Strahler et al., 2010).

However, unlike the martial artists, the CARs observed during the competition phase in the current study were not unchanged, but were significantly blunted compared with those during training. Furthermore, the competition phase and day of competition were accompanied by significantly greater levels of competition anxiety. Although no change in neuroendocrine reactivity in response to competition may be indicative of adaptation to competitive stress, the blunted responding and increased anxiety observed during the competition phase are more typical of chronic stress. Outside of sporting contexts, reduced CARs are signatures of fatigue, exhaustion, and burnout (Chida and Steptoe, 2009) and blunted CARs are typical in conditions of chronic stress, for example, in students experiencing academic stress (Lovell et al., 2011; Duan et al., 2013); informal caregivers (Mortensen et al., 2019); parents of children who display problem behaviors (Lovell et al., 2013); and following early life adversity (Mangold et al., 2010; Raffington et al., 2018). Blunted CARs have also been associated with impaired cortisol recovery following acute laboratory stress, demonstrating a link between deficits in these preparatory responses (Dienes et al., 2019). As increased training volume and competition can lead to higher levels of burnout, somatic stress, and reduced stress recovery in rowers (Jürimäe et al., 2002; Purge et al., 2005), the blunted CARs during the competition phase may therefore reflect burnout and exhaustion. The CAR is considered an adaptive response to prepare for forthcoming demands; a blunted CAR during competition is, therefore, not optimal in terms of performance and health. Indeed, it is suggested that training and competition loads that are too great can have a detrimental impact on performance (Purge et al., 2005). Moreover, blunted CARs are linked to a range of psychiatric and physical conditions (c.f. Fries et al., 2009), and prolonged hypo-responding is a risk factor for morbidity (Phillips et al., 2013). The follow-up period in the current study did not extend beyond the final competition weekend and, as such, longer term HPA activity and health status are unknown. As these rowers are young and healthy, a return to typical diurnal secretion of cortisol and a reduction in anxiety is expected; however, this is reliant on adequate recovery. Over-training, in the absence of adequate recovery can to lead to a plethora of negative health outcomes through physiological dysregulation. Such periods have been associated with a blunting of the CAR (Cadegiani and Kater, 2017) and higher levels of morning cortisol following a 4-week period of overload training in rowers (Smith et al., 2011), and this is exacerbated in the presence of psychosocial stressors (Meeusen et al., 2013). There is no specific marker of over-training in rowers, and in their review, Mäestu and Colleagues (2005) advocate an individualized approach to understanding impact of over-training in athletes. As sustained HPA dysregulation accompanied by high levels of anxiety places rowers at greater risk of psychological and physical morbidity, measurement of the CAR may provide a useful tool for assessing physical functioning during training and competition.

These findings should be considered in light of limitations. First, the sample size was dictated by the opportunity to sample a single crew of elite athletes; the current findings therefore warrant further investigation in a larger equivalent sample. Second, there were no objective measures of waking and sample provision and therefore protocol adherence is not known. This was, however, an extremely motivated cohort with strict training regimes which would necessitate typical waking times during the testing periods. Further, strategies which aim to increase the motivation to participate are associated with improved adherence. As such, in line with guidelines (Stalder et al., 2016), the researchers personally engaged the participants and their coach with the research goals, ascertained participant understanding of the procedures, and expressed the importance of adherence. Third, although there were no statistically significant differences between the weekends within each phase, there were differences in the nature of the two competitions. The first was the British Universities’ competition comprising eight crews, while the second was the inter-varsity race between two rival universities. Both competitions were significant events for the participating rowers; however, the importance of winning is far greater in an event with two teams. Furthermore, anecdotally, the inter-varsity race, between two local rival crews, attracts larger crowds that are closer to the crews, and is typically perceived as a higher stakes competition where reputation and pride are under more intense scrutiny. Critical social evaluation is a pertinent stressor that leads to HPA activation in acute situations (Dickerson and Kemeny, 2004). Indeed, in the context of competitive ballroom dancing, cortisol responses are greatest when being socially evaluated by judges, and these increases are over and above those attributed to the physical strain of exercise observed during training. Although no measures of perceptions of social evaluation were recorded in the current study, it is interesting to note that levels of cognitive and somatic anxiety, and awakening cortisol, were highest on the day characterized by greater social evaluation. Furthermore, in support of the notion that increased competition stress can have a detrimental impact on performance (Purge et al., 2005), and a blunting of the CAR is a maladaptive response in terms of preparation for demand, the crew lost this inter-varsity competition. It should be noted, however, that the perceived importance of each competition to the rowers was not formally ascertained. Finally, the CSAI-2 was used to assess perceived levels of cognitive and somatic anxiety prior to competition. Although frequently used to assess competition anxiety across a wide range of sporting contexts, concerns have been raised regarding its ability to distinguish between affective states (Jones and Uphill, 2004). For example, the same somatic symptoms can be attributed to excitement and fear (Kerr, 1997) leading to potential ambiguity in the meaning of the somatic anxiety sub-scale. Further, high scores on the cognitive anxiety sub-scale may not reflect cognitive anxiety per se, but moreover may reflect recognition of forthcoming challenge and the need to activate coping resources (Lane et al., 1999). Given the observation of blunted CARs and their association with fatigue, exhaustion, and burnout in non-sporting contexts, it is likely that, in these rowers, high scores represent higher levels of somatic and cognitive anxiety; however, the precise attribution of these perceptions is not known.

Notwithstanding these limitations, this study demonstrates clear differences in competition anxiety and HPA axis function between periods of training and competition in elite rowers. The observed increased anxiety and blunted CARs are not optimal for psychological and physical well-being or performance, and this warrants further investigation in a larger sample in order to consider strategies to facilitate more adaptive responding in elite rowers during competition.

## Data Availability

All datasets generated for this study are included in the manuscript and/or the Supplementary Files.

## Ethics Statement

This study was carried out in accordance with the recommendations of the “British Psychological Society” with written informed consent from all subjects. All subjects gave written informed consent in accordance with the Declaration of Helsinki. The protocol was approved by the Department of Psychology Ethics Committee at Northumbria University Newcastle.

## Author Contributions

MW designed and helped to execute the study, analyzed and interpreted data, and wrote the manuscript. DM designed and executed the study, recruited participants, analyzed and interpreted data, and contributed to the manuscript.

### Conflict of Interest Statement

The authors declare that the research was conducted in the absence of any commercial or financial relationships that could be construed as a potential conflict of interest.

## Supplementary Material

DATA SHEET 1 Diurnal cortisol and competition anxiety in elite rowers during training and competition.
